# Socioeconomic Characteristics Influencing Level of Awareness of Aflatoxin Contamination of Feeds among Livestock Farmers in Meru District of Tanzania

**DOI:** 10.1155/2018/3485967

**Published:** 2018-04-30

**Authors:** E. M. Ayo, A. Matemu, G. H. Laswai, M. E. Kimanya

**Affiliations:** ^1^The Nelson Mandela African Institution of Science and Technology, P.O. Box 447, Arusha, Tanzania; ^2^Sokoine University of Agriculture, P.O. Box 3004, Morogoro, Tanzania; ^3^Department of Rural Economy and Agriculture, African Union Commission, P.O. Box 3243, Addis Ababa, Ethiopia

## Abstract

Aflatoxins occurrence in feeds challenges human and animal health. Farmers' awareness status of these toxins has an effect on their level of exposure. The study assessed the influence of socioeconomic characteristics of farmers on their awareness of aflatoxin contamination of feeds. Data were collected from 258 households and analysed by SPSS program for descriptive statistics and association between socioeconomic characteristics and awareness of aflatoxin contamination of feeds. Over seventy percent of the farmers had never heard about aflatoxins. Education level, specialization, and period of keeping animals had significant influence on aflatoxin awareness. Hearing about aflatoxins was six times higher among farmers who studied life or social sciences than those without specialization and those who studied other fields. Awareness that aflatoxins may occur in feeds was twice higher among farmers with higher education than those with lower education. Perception that aflatoxins in feeds are detoxifiable was threefold higher among young people (with ≤10-year period of keeping animals) than among older ones. Awareness of aflatoxins was particularly low among farmers with low education and those without exposure to life or social sciences and vice versa. Sensitization is recommended to raise farmers' awareness on aflatoxin contamination of feeds and incorporating aflatoxin knowledge in school curricula.

## 1. Introduction

Aflatoxins are toxins occurring naturally as a result of fungal metabolism [1]. The toxins have been associated with various health problems in domestic animals and humans throughout the world [2, 3]. It is estimated that globally the region lying between 40°N and 40°S latitudes is generally at a risk of aflatoxin exposure through foods and feeds [2, 4]. Contaminated feeds are potential sources of chronic aflatoxins intake in human through foods of animal origin [5]. Almost all feed resources may contain aflatoxins when run mouldy [6]. The toxins have health hazards to animals and ultimately humans through transfer into animal products [7]. Various studies have reported ill impacts of aflatoxins in humans. These effects include immune suppression, liver cancer, digestive disorders, fertility impairment, and central nervous system interference [8]. Aflatoxins have similar effects to those caused by HIV/AIDS and may intensify susceptibility to HIV/AIDS [9].

In general, farmers and general public in developing nations know less about aflatoxins and the associated health impacts [4, 10]. A study conducted in Kenya showed farmers to perceive that eating mouldy food may be harmful, but they considered meat from animals fed mouldy feeds to be safe [11]. This shows that scenario of aflatoxin contamination of feeds is even less known. Studies done in many localities indicate that levels of awareness of aflatoxins are low. Some of the documented levels are, for example, 25% in Vietnam [12], 6% in Zimbabwe [13], 12% in The Greater Addis Ababa milk shed of Ethiopia [14], and 20% in Tanzania [15, 16].

Levels of awareness of aflatoxins and other fungal toxins have been found to vary with various socioeconomic characteristics. For instance, in Kenya, women were found more informed of danger of fungal toxins and cautious to mouldy feeds than men [11]. In Vietnam, young farmers (at age of 21–29) were found more informed of aflatoxins in crops than the older groups [12]. In Tanzania, researches [16, 17] have shown that education level has positive effect on aflatoxin awareness. In Ghana, it was [18] found that field of study particularly life sciences has positive impact on aflatoxin awareness. In Ethiopia, farmers were found less informed of aflatoxins than individuals in other occupations [19]. Scanty information is available on awareness of aflatoxins in relation to socioeconomic characteristics in many localities in Tanzania and other countries. Also the available reports are more deflected to awareness of aflatoxins in food crops such as ground nuts and maize than feeds. Where reports touching awareness on aflatoxins in feeds are available, they still lack some vital details that would be required for mitigation of challenges related to aflatoxins occurring in feeds. Moreover, the reports are less informative in terms of location specificity. Little is known about awareness of aflatoxin contamination of feeds among farmers based on socioeconomic characteristics, even in aflatoxin risky areas including the current study area. The situation may cause unknown levels of aflatoxin exposure to animals and humans and ultimately ruin the public health. Farmers' awareness in solving a farming problem may be considered as the first step towards identification and designing mitigation measures. Therefore, knowing the level of awareness of aflatoxins in feeds among livestock farmers is important in setting plans to reduce risks of aflatoxin exposure.

Studies show that, in intensive systems, including the study area, use of crop by-products such as maize bran and oil seeds as supplementary feeds is very high [20]. These by-products are the potential sources of aflatoxin exposure to animals; yet it is not well known whether farmers are aware of this scenario. The aim of this study was to assess the status of awareness by farmers on aflatoxin exposure through contaminated feeds. Information obtained from this study is useful in designing measures for increasing farmers' awareness towards reduction of aflatoxin exposure to livestock through contaminated feeds.

## 2. Methods

### 2.1. Description of the Study Area

The study was conducted in coffee-banana belt, Meru district, situated within 3.0°–3.4°S and 36.3°–37.0°E and altitude from 1200 to 1600 m a. s. l. on the slopes of mount Meru in Arusha region of Tanzania. The district experiences average annual precipitation and temperature of 1,200 mm and 25°C, respectively. The total population was 268,144, majority of them practicing mixed farming [21]. The district is among those experiencing intensive livestock keeping in Tanzania, particularly dairy cattle raising [22]. Owing to the intensification, the animals may be predisposed to aflatoxin hazards due to high feed supplementation with possible contaminated crop by-products [23].

### 2.2. Survey of Farmers Awareness and Socioeconomic Characteristics

A cross-sectional design using semistructured questionnaire was adopted for data collection of socioeconomic characteristics of respondents, which are gender, age, level of education, education stage, field of specialization, employment categories (formal and informal), occupation categories (farming and nonfarming), and length of being involved in keeping livestock. In this study, two levels of education were considered as low (<secondary education) and high (≥secondary education). Also two categories of academic specialization were considered. These were life/social sciences (farmers who studied science-based subjects in secondary education and those who got typical life or social science-based courses such as medical and agricultural courses) and none/other fields (farmers with low education with no academic specialization and those studied fields other than life and social sciences). Each farmer in the study sample was asked whether he/she had ever heard about aflatoxins and then was asked about his/her awareness in relation to the following items: (a) possibility of fungal toxins or aflatoxins to occur in feeds, (b) indicators for presence of fungal toxins or aflatoxins in feeds, (c) types of feed ingredients most prone to fungal toxin/aflatoxin contamination, (d) possibility of natural toxins in feeds to affect health of animals, (e) possibility of fungal toxins being transferred from feeds to foods of animal origin, (g) ability to identify/detect mould formation in feeds, and (h) whether fungal toxins or aflatoxins in feeds can be detoxified to render the feeds safe. Direct physical assessment was also made to ascertain some feed aspects in relation to farmer responses and views using detection indicators such as feed type, colour, odour, and consistence. The questionnaire was first prepared in English to retain the required context and then translated into Swahili for smooth face-to-face interview. It was then pretested, to check for its suitability, by administering it to twenty-five respondents in an area not included in the study as previously suggested [24]. Items noticed to be unclear in the questionnaire were legibly corrected.

### 2.3. Sampling Design and Sample Size Determination

Seven wards were purposively selected from thirty-five wards of the district based on the criteria of having higher population densities of livestock taking dairy cattle as reference.

Systematic random sampling technique was used to select households keeping livestock from the seven wards. The household sample size *n* amounting to 258 was obtained through the formula devised by Yamane [25] as follows:

(1) $$\begin{array}{c} n=\frac{N}{\left(1+Ne^{2}\right)}, \end{array}$$

where *N* is the sampling frame for households keeping livestock and practicing feed supplementation in the wards estimated to 725 as per district database; *e* is the acceptable sampling error of 0.05 at the 95% confidence level.

Household head, spouse, or any household member/employee with sound mind aged eighteen and above who declared to participate in the household livestock activities and is ready to play the part of household spokesperson was interviewed. Candidate wards with selected proportionate subsamples of households (in brackets) were Ambureni (35), Imbaseny (39), Nkoaranga (34), Patandi (38), Poli (38), Seela-Sin'gisi (42), and Songoro (32). All the information on livestock population size and distribution by households was obtained from Meru District Livestock Development office.

### 2.4. Data Analysis

Data were entered in EpiData 3.1 software for easy control of entry quality and then exported to Statistical Package for Social Sciences (SPSS) version 20 software for analysis. Descriptive analysis was carried out to obtain descriptive results, that is, frequency and percent distribution of the assessed variables from the data set. Bivariate regression analysis was preliminarily run to check for any crude association between the predictors and the outcome variables. Variables found to have any association were subjected to forward multivariate logistic regression to establish the actual significance and magnitude of association between the socioeconomic characteristics and awareness of aflatoxin contamination of feeds. A *p* value less than 5% was considered significant throughout the conducted analyses.

## 3. Results and Discussion

### 3.1. Socioeconomic Characteristics of Respondents

The targeted sample of household-respondents required for the study was well attained. Briefing was done such that prospect respondents were motivated to participate in the interview leading to very few household refusals in answering the questionnaire. However, the refusals were handled by utilizing the advantage of the adopted systematic sampling technique, which allows moving forward in selecting sampling units (households) until a required sample was obtained.

The socioeconomic characteristics of the respondents shown in Table 1 indicate that livestock farming as an economic activity is done by people of various social groups as was also reported by Amimo [26] in a survey done in Western Kenya. A little more men than women participated in the interview, a phenomenon related to the tendency that majority of the household heads are men. Farmer aged above 45 had higher proportion against the younger ones since the latter are likely to be more active away from homes. The sample had more farmers with high education than those of low education, probably due to the socioeconomic set-up in the area, such as resource scarcity, mainly land, that forces the majority to attain higher education as a coping strategy. Similarly, the number of famers with tertiary education (college and Universities) surpasses those with secondary education and less than secondary education. The reason is likely to be the same as for the low and high education categories of the farmers. Similar high education rate among farmers was previously reported by Nyangaga [27]. About half of all the respondents were found to have been exposed to life/social sciences based studies, a scenario reported elsewhere by Awuah et al. [18]. More farmers were found under formal employment than those under informal probably due to the tendency that most of the farmers have dual employment, formal being primary. Similar analogy is explained for the occupation in terms of farming and nonfarming. Proportion of farmers who have been keeping animals for ten years or less was a little bit more than those kept for over ten years. Possibly this is due to the tendency that more new people join the activity of animal keeping with time.

### 3.2. Description of the Perception of Respondents towards Feed Aflatoxins

The results on the descriptive analysis of the respondents' awareness of aflatoxin contamination of feeds are presented in Table 2. Only about a quarter of respondents had heard about the term aflatoxins. This level was relatively low as compared to the level of 93% reported by Marechera and Ndwiga [28] in Kenya and a bit higher than the value of 20% reported by Kamala et al. [15] in Kilosa district in Tanzania. The deviation may be due to time lag and locality attributes. For instance, in the Tanzanian cases, the study in Kilosa was conducted in 2010 and the current one was conducted in 2016 where different rates of awareness have been recorded. Other reasons may be due to factors such as nature of the study population. In Kenya, epidemiological events of aflatoxicosis that killed a number of people [29] might have raised louder alarm on aflatoxins. The fact that more than half of the respondents with awareness of aflatoxins got the information recently (≤1 year ago) compared to about one-fifth who got it about two years ago implies that there has been an increase in knowledge of aflatoxins with time in the study area. Over two-thirds of respondents who had ever heard about aflatoxins got the information from the mass media. The rest of respondents obtained the information through seminars and experts, neighbours and friends, and from written resources. Results show that just few farmers got information about aflatoxins through reading, probably indicating scarcity of written resources as information about aflatoxins, low reading motivation on the side of farmers, or else the materials being too technical for farmers. This implies that mass media may be the best way to sensitize livestock farmers, other key stakeholders, and the general public about aflatoxins and means to alleviate their exposure. By mass media in Swahili* vyombo vya habari *as mentioned by the farmers meant radio, television, and scantly newspapers which are the common and readily accessible sources of information. Of these, radio and television programs are considered the most appropriate sources of information especially for the Swahili-conducted programmes. Recently, there have been some initiatives to inform the public about aflatoxins in Tanzania through radio, television, and newspapers [30]. Perhaps the current level of awareness is the result of the initiatives in the country.

Distribution of respondents based on having information about aflatoxins and socioeconomic characteristic is shown in Table 2. Farmers with socioeconomic characteristics of higher education, college education, life/social sciences, formal employment, nonfarming occupations, and Imbaseny locality showed relatively higher knowledge of aflatoxins than their counterparts. The education level and field of specialization seemed to be the major socioeconomic factors governing farmer's awareness of aflatoxin contamination of feeds. For instance, there was logical increase of proportion of farmers with knowledge of aflatoxins with advancing stages of education. Influence of high education and academic exposure to life or social sciences in promoting awareness of aflatoxin contamination was also reported by other researchers [16, 17, 31]. The implication is that education level has direct and indirect (through other socioeconomic characteristics such as employment and occupation) positive influence on awareness of aflatoxin contamination. Other minor promoters of awareness of aflatoxin contamination of feeds were female gender, young age, and short period of keeping animals. The finding that short period of keeping animals is associated with awareness about aflatoxins was not expected. Probably women are more engaged in managing livestock than men while young farmers are likely to be in the educated group, able to access information faster. Short period of keeping animals may also be linked with young age which again favours information access on various issues including animal keeping activity.

Among the wards, Imbaseny ward followed by Nkoaranga ward showed relatively higher proportions of farmers with information about aflatoxins. Geographically these wards engulf more number of academic institutions including higher learning institutions compared to the other wards such as Ambureny which are a bit in remote area far from these institutional centres. The institutional set-up seems to have attracted a number of educated people, some of them also keeping animal, and their broader knowledge is likely to influence awareness of aflatoxin contamination of feeds.

The descriptive statistics on the farmers' awareness on the general fungal toxins are shown in Table 3. About half of the respondents were aware that feeds may contain natural fungal toxins. Those found aware were further asked to mention by name any specific fungal toxins that may occur in feeds. Of these, only few (6%) managed to come out with the term “aflatoxins” or its translation as* sumu-kuvu* in Swahili (the communication media used in the interview). Majority could not name any specific fungal toxins though they perceive that feeds may contain some inherent toxins on spoilage. About 19.5% managed to give at least miscellaneous and broader concepts as they perceive, such as mould, mould toxins/products, cancer causing toxins, diarrhoea-causing toxins, bloat-causing toxins, feed/food mould, and toxins due to rotting/spoilage/rusting. In a similar study by Jelliffe et al. [32], respondents had difficulty in naming the toxins occurring in groundnuts as “aflatoxins”; instead they called them mould or bitter nuts. The important fungal toxins known to occur in foods and feeds include aflatoxins, fumonisins, trichothecenes, zearalenone, citrinin, ergot alkaloids and ochratoxins A, and patulin, aflatoxins being the most hazardous toxins [33]. The result implies that livestock farmers have limited information about the fungal toxins and aflatoxins in particular. The low awareness and unclear concept about aflatoxins is common in many settings as reported in other studies [13–15, 33]. The situation may allow high aflatoxin exposure level through contaminated feeds leading to health hazards to animals and humans.

Majority of the farmers (Table 3) perceived that maize bran is the most susceptible feed ingredient to fungal toxin formation. This is supported by another report [23] that maize is one of the most susceptible cereals to mycotoxin contaminations. Maize genome has genes that easily encode formation of some enzymes favouring fungal growth, sporulation, and toxin production and additionally offer little environmental stress resistance which predisposes plants to toxigenic fungal invasion [34]. In addition, bran as by-products of cereal grains is the major sinks of mycotoxins initially carried in the whole grains [35]. Some farmers perceived that moisture in the maize bran due to water sprinkled into the maize grain prior to or during dehulling process favours further growth of the toxigenic mould with time in storage. One of the respondents, also a corn miller, commented that water added in maize for dehulling and heat generated cause the bran spoilage and eventual toxin formation if quick drying of bran is not done. In Tanzania, where dry milling is a common practice, the farmers' perception that maize bran is the most susceptible to fungal contamination is valid. Feed spoilage and contamination may occur due to relatively high postharvest moisture content, improper drying, delayed drying, and storage with moisture above critical values for mould growth [36, 37]. This calls for prompt and proper drying of feeds, particularly maize bran as a supportive measure in alleviating exposure to aflatoxin contamination of feeds.

A number of the respondents (Table 3) perceived that feeds with fungal toxin contamination have health hazards to animals. Empirical evidences support the perception [5, 38]. With acute levels such toxins may be fatal within short time while chronic levels may cause death after a relatively long time through immunosuppression, encouraging vulnerability and opportunistic diseases [39]. The fungal toxin contamination of feeds is also associated with animal production loss due to the impaired health leading low production performance [23]. High proportion of the respondents (Table 3) had opinion that natural feed toxins cannot be transferred to animal tissues and ultimately to the foods of animal origin. The findings concurred with another report [11] which showed perception of some dairy farmers in Kenya that direct eating of mouldy food is harmful but eating products from animals fed mouldy feeds is safe. Other reports [23, 40] refute this perception. Their studies showed that fungal toxins consumed in feeds by animals are assimilated into body tissues and then released into foods of animal origin as metabolites of the original toxins, which are also toxic to the secondary consumers. Studies have shown that aflatoxin B1 is metabolized to aflatoxin M1 (AFM1) in the liver and then transferred to milk, eggs, and meat of animals which ingested the toxin in feeds. Independent studies have been done by different researchers to validate this perception [6, 41–43]. Residues of aflatoxins were found in raw cow milk [6], eggs [42], and broiler meat [41–43]. The amount of AFM1 in fresh milk may range from one to seven percent of the total amount of aflatoxin B1 ingested in a diet [5]. In higher-yielding animals consuming large amounts of concentrates, the transfer rate from feeds to milk may be higher. Aflatoxin transfer to eggs and chicken meat has been found at rates of 0.1% and 0.01%, respectively [5]. These levels of aflatoxin transfer to foods of animal origin make a chronic intake of the toxins to human leading to great health risk. In practical sense these technicalities are beyond the knowledge capacities of many farmers and therefore there is a need to simplify them into simpler expirations to suit all farmers. With this, farmers can comprehend the problem of these toxins in food chain, on top of what they know about mouldy feeds that they have health hazards in animals only and no transfer to foods of animal origin. This will build care and habit among farmers to avoid feeding mouldy feeds to animals.

The respondents reported that they suspect presence of fungal toxins in feeds if the feeds look spoiled and may be tested by one or more of the indicators shown in Table 3. Feed abnormal colour such as brownish, blackish greenish, or bluish, rotten or soil smell, abnormal consistence such as clumps and fibrous forms, and presence of insect larvae were reported as key indicators for quick tests of feed spoilage. Other indicators to suspect presence of fungal toxins in feeds according to the farmers were, for example, animal refusals of the feeds especially if associated with abnormal smell, general poor appetite of animals, abnormal milk taste, poor health, and animal deaths. These indicators and symptoms were also reported in an on-farm study [9] on strategies to manage mould and fungal toxin formation in feeds. When strictly and carefully utilized, the indicative signs and symptoms may be helpful in detecting mouldy feeds that are likely to be contaminated with aflatoxins. However, it is worth noting that absence of these signs does not guarantee that the feeds are entirely free of the toxins and safe. Studies have shown that it is very difficult to have feeds free of fungal toxins under normal environment. According to these results, feed discoloration and off-smell are useful frontline indicative factors to suspect feed contamination and possibly presence of aflatoxins and other fungal toxins. Some respondents declared not knowing any indicator to suspect presence of these toxins in feeds. Inability to suspect and detect feed spoilage and contamination using quick test may allow exposure to aflatoxin contamination of feeds thus putting consumers into higher health risk. High proportion of the respondents declared that they know and are able to detect mould formation in feeds (Table 3). This is because though fungal toxins in feeds are not visible, moulds growing on feeds are visible. The farmers reported that moulds often colour and affect the appearance of the feed on which they are growing [9]. Feeds invaded with mould take on an unappealing/off smell. It is well known that presence of mould in feeds is a good indicator of possible contamination with fungal toxins which may help the farmer in deciding to discard the feeds. About two-thirds of the respondents perceived that fungal toxins already formed in feeds can be detoxified to render the feed safe for animal. The respondent reported that possibly soda-ash, plant ashes, charcoal, salt, and some herbs may reduce the fungal toxins if fed with feed resources suspected to be contaminated. Ashes are used in treating animal feeds for other purposes such as reducing ant nutritional factors in monogastric animals [44] and fibre digestibility improvement [45]. Some compounds in form of antioxidants from plants sources have counteractive effect against the oxidative stress induced by aflatoxin in animal body after absorption [46].

Individuals with social sciences based specialities are likely to be more socially interactive and curious to get information on many life issues including health alarms such as aflatoxin hazards. Life scientists are concerned with function and interactions of living organisms and their environments and social scientists with society and social life, engineers are more concerned with designing, constructing, and testing structures, materials, and systems. Educational background and interest may cause significant variation in levels of awareness of aflatoxin contamination of feeds among livestock farmers. About forty-two percent of the farmers who attained higher education and studied life or social sciences were found aware of aflatoxins while only about twenty-five percent of those who attained higher education and studied other fields were aware of aflatoxins (Table 4). This disparity may be due to the effect of academic specialization which is likely to favour or disfavour interest and curiosity towards issues such as contamination of feeds and associated hazards.

### 3.3. Association of Socioeconomic Characteristics and Awareness of Aflatoxin Contaminated Feeds

Tables 5 and 6 show crude and adjusted associations between some socioeconomic characteristics and awareness of aflatoxin in contaminated feeds, respectively. The results show that likelihood of having heard about aflatoxins was six times higher among farmers who studied life and social sciences compared to those with no specialization and those who studied other fields. The finding matched to another report [18] which found a similar analogy of aflatoxin awareness menace in Ghana. Probably individuals who studied life sciences are capable of recalling and accessing information related to microbiology/mycology in which fungal products are studied.

Farmers with higher level of education (≥secondary education) were twice more aware that aflatoxins do occur in feeds than those with lower education. This result concurs with finding of other studies showing that people with higher education have higher chances to be informed and more aware of risky factors in food than people with less education [16, 17, 31]. In another similar analogy, [27] found that people with secondary and tertiary education were more aware of aflatoxins in foods and feeds than those of lower education. This may be linked to the general high reasoning capacity of the learned people.

Livestock farmers under formal employment were five times likely to be able to detect mould formation in feeds than those under informal employment. The reason may be due to the tendency that majority of the individuals under formal employment are those with higher education. Additionally they are likely to have close contact and wider chance of sharing information and experience with each other on various issues that may include news on aflatoxins. Likelihood of knowing that aflatoxins contamination of feeds is detoxifiable was three times higher among farmers who kept animals for ten years or less compared to those who kept animals over ten years. The relationship between short time of keeping animals and more awareness may be linked to young age status of the farmers. Young individuals are likely to be learned with broader reasoning capacity that potentially supports the perception. This is also supported by the observation that higher proportion of young farmers had heard about aflatoxins compared to older ones (Table 3) as similarly reported elsewhere [12].

## 4. Conclusion and Recommendation

Majority of the livestock farmers have never heard about aflatoxins. Higher education at least at secondary school level and exposure to life or social sciences impart positive effect on awareness of aflatoxin contamination of feeds among livestock farmers. Short experience of keeping animals has more promoting effect on farmers' awareness that feed aflatoxin can be detoxified to reduce exposure. It is therefore concluded that awareness of aflatoxin contamination of feeds among livestock farmers in the study area is generally low especially among farmers with low education and those lacking life or social science exposure. It is recommended that authorities overseeing food and feed safety should sensitize livestock farmers on the aflatoxins, preferably through extension services to safeguard public health from exposure to aflatoxin contamination of feeds. As a long term measure, the Government should introduce aflatoxin issues in curricula for primary and secondary schools as well as agriculture and health colleges to increase access of knowledge to aflatoxin.

## Figures and Tables

**Table 1:** Socioeconomic characteristics of the respondents (*n* = 258).

Socioeconomic characteristics	Categories	Frequency (%)
Gender	Female	119 (46)
	Male	139 (54)

Age	≤45 years	114 (44)
	>45 years	144 (56)

Education level	Low education level	110 (43)
	High education level	148 (57)

Education stage	<secondary education	110 (43)
	Secondary education	31 (12)
	Tertiary education	117 (45)

Field of specialization	None, general/engineering sciences	126 (49)
	Life/social sciences	132 (51)

Employment category	Formal	111 (43)
	Informal	147 (57)

Occupation type	Farming	124 (48)
	Nonfarming	134 (52)

Animal keeping experience	≤10 years	132 (51)
	>10 years	126 (49)

**Table 2:** Distribution based on having heard about aflatoxins with respect to socioeconomic characteristics (*n* = 258).

Socioeconomic characteristics	Categories	*n*	Heard about aflatoxins	
			Frequency	% of *n*
Gender	Female	119	39	33
	Male	139	32	23

Age	≤45 years	114	35	30
	>45 years	144	36	25

Education level	Low education level	110	12	11
	High education level	148	59	40

Education stage	<secondary education	110	12	11
	Secondary education	31	8	26
	College/tertiary	117	51	44

Field of specialization	Life science and social sciences	132	55	42
	None, others (general/engineering sciences)	126	16	13

Employment category	Informal	147	24	16
	Formal	111	47	42

Occupation	Farming	124	18	15
	Nonfarming	134	53	40

Period of keeping animals	≤10 years	132	44	33
	>10 years	126	27	21

Location (wards)	Ambureny	35	4	11
	Imbaseny	39	22	56
	Nkoaranga	34	13	38
	Patandi	38	9	24
	Poli	38	9	24
	Seela-Sing'isi	42	8	19
	Songoro	32	6	19

**Table 3:** Awareness distribution on fungal toxins contamination of feeds.

Respondents' perceptions on feed aflatoxins	Frequency (%)
*Possible presence of fungal toxins in feeds (n = 258)*	
Yes	133 (52)
No	111 (43)
Not certain	14 (5)
*Specific probable fungal toxins in feeds (n = 133)*	
Aflatoxins	8 (6)
Other toxin fungal names	26 (20)
Do not know	99 (74)
*Feed ingredient susceptible to fungal toxin contamination (n = 133)*	
Maize bran	96 (72)
Wheat feeds	3 (2)
Wheat pollard	5 (4)
Sunflower seed cake	1 (1)
Cotton seed cake	1 (1)
Other feed ingredients	4 (3)
Do not know	23 (17)
*Possibility that fungal toxins in feeds affect animal health (n = 133)*	
Yes	113 (84)
Not certain	18 (14)
No	2 (2)
*Possibility that fungal toxins are transferred from feeds to foods of animal origin*	*(n = 133)*
Yes	21 (16)
Not certain	11 (8)
No	101 (76)
*Signs to suspect presence of fungal toxins in feeds (n = 133)*	
Abnormal colour	66 (48)
Abnormal consistence	24 (18)
Bad odour (rotten/soil smell)	47 (36)
Insect/larva presence	3 (2)
Impaired animal health/deaths	13 (5)
Do not know any indicator	24 (18)
*Ability to detect mould in feeds (n = 133)*	*133*
Yes	123 (93)
No	9 (7)
Not certain	1 (1)
*Possibility of detoxifying aflatoxins in feeds (n = 133)*	*133*
Yes	83 (62)
No	38 (29)
Not certain	12 (9)
*Heard about aflatoxins (258)*	
Yes	71 (28)
No	187 (72)
*Means through which aflatoxins were heard (71)*	
Reading	3 (4)
Mass media (radio/TV)	49 (69)
Seminars/experts	11 (16)
Friends/neighbours	8 (11)
*Time when heard about aflatoxin(s)*	
≤one year ago	40 (56)
Two years ago	15 (21)
>two years ago	16 (23)

**Table 4:** Respondent distribution by levels of education, field of specialization, and awareness about aflatoxins.

Level of education	Specialization/means of accessing information	*n*	Heard about aflatoxins
			Frequency (% of *n*)
	*Specialization*		

Low	Life/social sciences	0	0 (0)
	None/other fields	110	12 (11)

High	Life/social sciences	132	55 (42)
	None/other fields	16	4 (25)

Total		258	71 (28)

	*Means of accessing information*		

Low	Reading	3	1 (33)
	Mass media (radio/TV)	69	6 (9)
	Seminars/experts	11	1 (9)
	Friends/neighbours	8	3 (37)

High	Reading	3	2 (67)
	Mass media (radio/TV)	69	43 (91)
	Seminars/experts	11	10 (91)
	Friends/neighbours	8	5 (63)

**Table 5:** Crude association between socioeconomic characteristics and feed aflatoxin awareness variables.

Socio-economic characteristics	Having heard about aflatoxin(s)		Perceiving aflatoxin presence in feeds		Being able to detect feed mould		Perceiving aflatoxin detoxification in feeds	
	COR (95% CI)	*p* value	COR (95% CI)	*p* value	COR (95% CI)	*p* value	COR (95% CI)	*p* value
*Gender*								
Female	2.1 (1.1–4.2)	0.04	1.0 (0.6–1.7)	0.87	1.2 (1.3–4.2)	0.82	2.1 (1.0–4.3)	0.42
Male (r)								

*Respondents' age*								
≤45 yrs	1.1 (0.6–2.1)	0.81	1.5 (0.9–2.4)	0.12	4.6 (1.0–22.7)	0.06	1.0 (0.9–3.7)	0.10
>45 yrs (r)								

*Level of education*								
High	6.4 (2.8–14.9)	0.00	2.0 (1.2–3.3)	0.01	2.2 (0.5–11.0)	0.32	1.4 (0.7–2.9)	0.40
Low (r)								

*Stage of education*								
≤secondary (r)								
Secondary	3.3 (1.0–10.7)	0.05	2.0 (1.2–3.4)	0.01	2.9 (0.6–14.3)	0.19	1.6 (0.7–3.5)	0.24
Tertiary	7.7 (3.2–18.6)	0.00	1.7 (1.1–2.8)	0.03	4.1 (0.8–19.9)	0.08	1.7 (0.8–3.4)	0.15

*Field of specialization*								
Life/social sc.	5.8 (2.7–12.60)	0.00	1.8 (1.1–2.9)	0.03	1.8 (0.4–7.2)	0.44	1.3 (0.6–2.7)	0.46
None/other fields (r)								

*Employment category*								
Formal	4.7 (2.2–9.7)	0.00	1.5 (0.9–2.5)	0.09	4.8 (1.0–23.5)	0.05	2.5 (1.2–5.1)	0.14
Informal (r)								

*Occupation*								
Farming (r)								
Nonfarming	4.3 (2.0–9.1)	0.00	1.6 (1.0–2.7)	0.05	3.1 (0.6–15.4)	0.16	2.3 (1.1–4.8)	0.03

*Animal keeping experience*								
≤10 yrs	1.4 (0.7–2.8)	0.35	1.9 (1.1–3.1)	0.01	1.7 (0.4–6.9)	0.45	2.9 (1.4–6.3)	0.01
>10 yrs (r)								

*Location (wards)*								
Ambureni (r)								
Imbaseny	7.0 (2.0–25.0)	0.00	0.2 (0.1–0.55)	0.00	2.8 (0.3–22.8)	0.34	1.7 (0.4–7.0)	0.48

COR: crude odd ratio; (r): reference.

**Table 6:** Adjusted association between socioeconomic characteristics and feed aflatoxin awareness variables.

Socioeconomic characteristics	Having heard about aflatoxin(s)		Perceiving aflatoxin presence in feeds		Being able to detect feed mould		Perceiving aflatoxin detoxification in feeds	
	AOR (95% CI)	*p* value	AOR (95% CI)	*p* value	AOR (95% CI)	*p* value	AOR (95% CI)	*p* value
*Gender*								
Female	1.6 (0.7–3.5)	0.27	1.1 (0.6–1.8)	0.82	1.1 (0.3–4.3)	0.93	1.6 (0.7–3.5)	0.23
Male (r)								

*Respondents' age*								
≤45 yrs	1.6 (0.6–4.1)	0.36	1.1 (0.6–2.0)	0.74	4.1 (0.6–27.6)	0.15	1.1 (0.4–2.7)	0.83
>45 yrs (r)								

*Education level*								
High	1.5 (0.3–8.2)	0.65	2.0 (1.2–3.3)	0.01	1.4 (0.6–3.2)	0.50	1.4 (0.2–8.0)	0.72
Low (r)								

*Education stage*								
≤secondary (r)								
Secondary	1.6 (0.3–7.7)	0.53	2.4 (0.6–10.0)	0.21	1.0 (0.01–93.5)	0.93	3.2 (0.3–33.6)	0.32
Tertiary	2.4 (0.4–17.0)	0.37	1.1 (0.4–3.3)	0.85	1.9 (0.8–4.5)	0.13	2.3 (0.3–16.0)	0.38

*Field of specialization*								
Life/social sc.	5.8 (2.7–12.6)	0.00	1.3 (0.4–4.0)	0.66	1.4 (0.1–1.5)	0.77	1.3 (0.3–6.0)	0.71
None/other fields (r)								

*Employment category*								
Formal	1.2 (0.2–6.8)	0.81	1.3 (0.4–3.9)	0.62	4.8 (1.0–23.5)	0.05	5.8 (0.8–43.7)	0.09
Informal (r)								

*Occupation category*								
Farming (r)								
Nonfarming	1.9 (0.5–6.8)	0.33	1.1 (0.5–2.50)	0.79	1.2 (0.1–22.5)	0.91	1.0 (0.3–3.8)	0.94

*Animal keeping experience*								
≤10 yrs	1.4 (0.5–3.8)	0.50	1.5 (0.8–2.8)	0.16	1.6 (0.3–10.3)	0.60	2.9 (1.4–6.3)	0.01
>10 yrs (r)								

*Location (wards)*								
Ambureni (r)								
Imbaseny	6.0 (1.4–25.5)	0.01	0.3 (0.1–1.2)	0.09	0.4 (0.1–2.5)	0.31	1.9 (0.4–7.0)	0.48

AOR: adjusted odd ratio; (r): reference.
